# A simple method to test geometrical reliability of digital reconstructed radiograph (DRR)

**DOI:** 10.1120/jacmp.v11i1.3128

**Published:** 2010-01-29

**Authors:** Stefania Pallotta, Marta Bucciolini

**Affiliations:** ^1^ Dipartimento di Fisiopatologia Clinica Universita’ di Firenze Firenze Italy

**Keywords:** image registration, treatment planning system, DRR

## Abstract

In radiotherapy treatment, portal images and digitally reconstructed radiographs (DRRs) are the most frequently used image‐guidance systems to monitor patient setup during clinical routine. DRR is therefore a critical element in the process of virtual simulation and patient position verification in radiotherapy. The aim of this work was to assess the geometric reliability of the DRR generated by XiO, Pinnacle, Odyssey and Oncentra Masterplan treatment planning systems (TPS). Instead of the computed tomography (CT) images of a quality assurance phantom, two synthetic phantoms were created and used to asses the linearity and the ray tracing reliability of the DRR generators. The obtained results demonstrate that the linearity and the reliability of DRR generators was less than 1 pixel for all TPSs.

PACS numbers: 87.55.D, 87.57.cp,87.57.nf

## I. INTRODUCTION

In conformal radiotherapy treatment, patient setup is a crucial point as the patient position registered in the computed tomography (CT) images used by treatment planning system (TPS) must be reproduced during each treatment session. Setup is routinely verified by comparing portal images with a reference image which records the intended patient position. As a reference, both simulator images and digitally reconstructed radiographs (DRR) can be used^(1)^ but in order to limit the onset of adjunctive error, the simulator step is often skipped and the DRR is widely recognized as the reference image.^(2)^

DRRs are critical elements in the process of patient position verification. In fact, when portal images are registered on DRRs, the geometrical accuracy of both images must be guaranteed in order to trust the registration results and use these data to correct patient setup. Deformations of reference and/or match images have a direct effect on the global accuracy of registration algorithms based on rigid transformations. The reliability of the ray tracing procedure and the linearity of the produced images have, therefore, to be investigated in DRR images.^(^ ^3^ ^,^ ^4^ ^)^ Despite the reliance on DRR images for both treatment design and verification, little work has been done to quantify the geometrical accuracy of DRRs.^(5)^ Two phantoms^(^ ^6^ ^,^ ^7^ ^)^ containing test patterns were developed and used to evaluate image quality and geometrical accuracy of DRR.

This study aims to present a method which, instead of real CT data, uses computerized phantoms to test the geometrical reliability of the DRR generators embedded in XiO, Pinnacle, Odyssey, and Oncentra Masterplan treatment planning systems (TPS). By using a computerized phantom, it is possible to eliminate from the analysis the contribution of pixel size and slice thickness, thus providing opportunity to evaluate the actual DRR generator's performances.

## II. MATERIALS AND METHODS

### A. Synthetic phantoms

**Figure 1:**
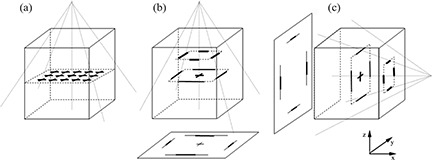
(a): The synthetic phantom for DRR linearity test is a homogeneous cuboid with a regular grid of crosses lying on the central coronal plane; (b, c) two representations of the synthetic phantom for the DRR ray tracing test; four couple of lines lying on coronal and sagittal planes are represented separately for demonstration purposes only. Two sketched views of the resulting DRR are also reported, together with the beam geometry used for DRR generation.

In order to test the geometric accuracy of DRRs considering, in particular, the reliability of the ray tracing procedure and the linearity of produced images, two synthetic phantoms were created (Fig. 1). The sampling used to acquire CT data affects DRR image quality and, if the voxel is not cubic (as is usual in CT data), the spatial resolution of DRR is not the same along the two directions. Although this effect compromises DRR image quality, it is independent from the DRR generators. The use of a computer generated phantom, enabling the elimination of the contribution of pixel size and slice thickness, allows one to concentrate the analysis on the geometrical accuracy of DRR generators.

The first phantom consists of a homogeneous cuboid containing a regular grid of forty‐five crosses on the central coronal plane; the second is a homogeneous cuboid containing couples of lines lying on parallel coronal and sagittal planes. The thickness of each line or cross is one pixel. Corresponding lines lying on parallel planes are built in order to overlap when beam divergence effects are considered. In order to generate realistic DRRs, the Hounsfield values of soft tissue and cancellous bone was assigned to cuboids, crosses and lines pixels of both phantoms. The reference system was chosen according to IEC61217 patient coordinate system.^(8)^ The two synthetic studies (160 slices, 512×512 pixels; 1 mm slice thickness and 1×1mm pixel size) generated using Analyze (BIR, Mayo Clinic, Rochester, MN) were converted in DICOM format and sent to the TPSs using the standard DICOM protocol.

### B. DRR

DRR images were generated using four TPSs: XiO (CMS, St. Louis, USA), Pinnacle (Philips. Radiation Oncology Systems, CA, USA), Odyssey 3.6 (PerMedics Inc, CA, USA), and Oncentra Masterplan 1.4 (Nucletron, The Netherlands).

All the DRR generators use a volume rendering technique that consists of simulating X‐rays passing through the reconstructed CT volume based on the absorption only optical model, thus generating an X‐ray like image. All the TPSs offer several options to optimize image quality, enabling the user to choose the appearance of the produced image. The parameters provided by the considered systems are different, often TPS‐related and, therefore, difficult to compare. In all cases, the CT to electron density table used for dose computation was also used for DRR generation, which means that no threshold or filtering was applied to the original data.

Window and level values resulting in optimal DRR image contrast were chosen in each TPS. For example, Pinnacle generates DRRs on 512×512 matrix, 8‐bit pixel depth, using a ray casting algorithm that provides three methods of interpolation. The DRRs, used for this test are generated using the trilinear interpolation algorithm. Odyssey uses a ray casting algorithm and a neighborhood interpolation method to generate 512×512DRRs, 16‐bit pixel depth. In XiO, two DRR options are available: normal and enhanced mode. The second option, retaining the CT voxel size dimensions and setting the dynamic range to 16‐bit, produces higher image resolution, and this option was used in this study. Finally, in Oncentra Masterplan the DRR image resolution and the dynamic range are variable; in this study, we used a 512×512 matrix and 16‐bit pixel depth.

For the linearity test, the isocenter of an anterior‐posterior (AP) beam was positioned on the central cross of the synthetic phantom, and a DRR for each TPS was generated.

For the ray tracing test, two orthogonal beams were positioned on the central cross of the second synthetic phantom and two orthogonal DRRs for each TPS were created. The DRR generators were tested checking the overlap of corresponding lines’ projection in the AP and left‐right (LR) DRRs. Errors in the ray tracing algorithm will manifest as shifts in the lines with respect to one another in the DRR images. The phantom was built in order to specifically test the two orthogonal projections conventionally used for patient positioning verification.

The DRRs were saved in DICOM format and sent to Analyze where the positions of the forty‐five crosses were sampled. The obtained results were compared with the a priori known data.

## III. RESULTS

**Figure 2:**
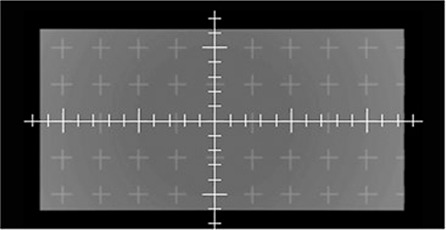
DRR of the synthetic phantom created to test the linearity of DRR generators. The image, shown here as an example, was obtained using Pinnacle TPS.

An example of the regular grid of crosses, visible in all DRRs, is reported in Fig. 2.

**Figure 3:**
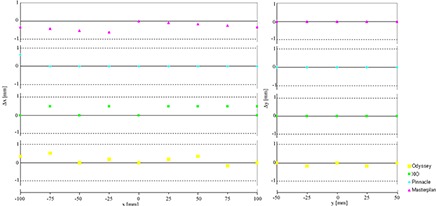
DRR linearity test; differences (Δx, Δy) between the registered and the theoretical cross's position plotted against x and y cross's coordinates.

The results of the linearity test are described in Fig. 3, in which the differences (Δx and Δy) between the positions of the forty‐five crosses in the DRRs and the a priori known values are plotted against the coordinates of the x and y crosses.

In the DRRs generated using Pinnacle and Odyssey TPSs, the measured differences Δy and Δx are almost the same, while a slight different behavior is observed for XiO and Oncentra Masterplan. It is useful to observe that the rendering process enlarges the crosses, making them visible in two‐five pixels and that the DRRs produced by different TPSs have a slightly different aspect due to the different implementation and interpolation strategies used. In Pinnacle and Odyssey, the DRR appearance is more smoothed then in XiO and Oncentra MasterPlan (even if a smoothing filter was used). In these cases the crosses are visible in three‐five pixels and the central pixel, having the higher pixel value, was chosen to represent the position of each cross. In the other cases, when only two pixels are involved, the crosses’ positions were likewise chosen in correspondence of the higher pixel value, causing 1 pixel shift in the localization of some crosses. In any case, the obtained differences are always less than 1 mm (1 CT voxel side) as seen in Table 1, where maximum and mean differences between measured and a priori known values along the x‐ and y‐axes are reported.

**Table 1:** DRR linearity test: $M_{x},\;M_{y},\;-m_{x},\;-m_{y},\;\sigma (m_{x}),\;\sigma (m_{y})$ represent the maximum value, the mean value, and the standard deviation of the differences between the theoretical and measured crosses position along x‐ and y‐axis, respectively.

*TPS*	$M_{x}\;[\text{mm}]$	${\bar{m}}_{x}\;[\text{mm}]$	$\sigma (m_{x})\;[\text{mm}]$	$M_{y}\;[\text{mm}]$	${\bar{m}}_{y}\;[\text{mm}]$	$\sigma (m_{y})\;[\text{mm}]$
MasterPlan	0,59	0,30	0,19	0	0	0
Odyssey	0,52	0,19	0,18	0,17	0,07	0,09
Pinnacle	0,66	0,07	0,21	0	0	0
XiO	0,50	0,32	0,24	0	0	0

**Figure 4:**
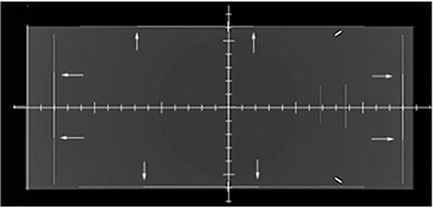
DRR of the synthetic phantom created to test the ray tracing accuracy of DRR generators; white dotted arrows point overlapping lines. The image was obtained with XiO TPS.

As for the ray tracing test, corresponding couples of lines are overlapped in both AP and LR DRRs originating from all TPSs, thus proving the reliability of the ray tracing procedures considered. As an example, one of the DRR obtained is reported in Fig. 4.

## IV. DISCUSSION

In many radiotherapy clinics, geometric patient setup uncertainties are measured and corrected registering two orthogonal DRRs on corresponding portal images. When such an approach is followed, the accuracy of patient setup depends also on the reliability of DRR images. The aim of this work was to present a simple method to test the geometrical accuracy of DRR using synthetic phantoms instead of real CT data. The linearity of the DRR images and the reliability of the ray tracing algorithms used for DRR generation were tested in XiO, Pinnacle, Odyssey, and Oncentra MasterPlan TPSs, using two synthetic phantoms specifically designed and created.

The results obtained demonstrate the linearity of all the considered DRRs: all the differences between measured and a priori known values are less than 1 mm, namely the CT study voxel side. The different interpolation and smoothing strategies used to generate the DRRs are responsible for slight differences among the TPSs. The ray tracing procedure was demonstrated to be reliable for all the TPSs as the two couple of lines result overlapped in both the AP and LR DRRs.

## V. CONCLUSIONS

The synthetic phantoms created and used to asses the performances of DRR generators demonstrate that the linearity and the ray tracing reliability of XiO, Pinnacle, Odyssey, and Oncentra Masterplan were less than 1 pixel. Moreover, the file format used for both phantoms allows their employment on each DRR generator DICOM compliant.

## ACKNOWLEDGEMENTS

The author would like to thank Dr. Francesco Meucci and Dr. Lucia Paladini for their help in collecting data, and Jay Natelle for carefully reviewing the manuscript.
